# 单中心BCL-2抑制剂治疗伴t(11;14)的复发/难治性多发性骨髓瘤患者疗效及安全性分析

**DOI:** 10.3760/cma.j.issn.0253-2727.2022.02.010

**Published:** 2022-02

**Authors:** 静怡 毕, 磊 温, 文冰 段, 扬 刘, 莎莎 王, 晓军 黄, 瑾 路

**Affiliations:** 北京大学人民医院血液科，北京大学血液病研究所，国家血液系统疾病临床医学研究中心，北京 100044 Department of Hematology, Peking University People's Hospital, Peking University Institute of Hematology, National Clinical Research Center for Hematologic Disease, Beijing 100044, China

多发性骨髓瘤（MM）是浆细胞异常克隆增殖的恶性肿瘤，是目前血液系统的第二大肿瘤，中国大陆地区发病率为1.15/10万[1]。近20年来，MM治疗领域不断进步，但是MM仍不可治愈。对于难治复发患者仍需要新的药物治疗，现有的蛋白酶体抑制剂及免疫调节剂均为泛靶向治疗药物，而维奈克拉作为一种高选择性BCL-2抑制剂，是首个依赖可靠生物学标志的分子靶向药物，其出现标志着MM的治疗进入真正意义上的精准治疗。目前，对于伴t（11;14）或BCL-2高表达的复发难治性MM（relapsed/refractory, RRMM）患者，维奈克拉Ⅲ期临床试验显示其总体有效率（ORR）达90％[2]，但国内尚无关于BCL-2抑制剂治疗MM的相关研究报道。为了解该类药物在中国患者中的疗效及安全性，本研究分析了本院收治的伴t（11;14）的以维奈克拉为主治疗方案的17例MM患者临床资料，探讨该类药物治疗MM的疗效及安全性，是国内首个BCL-2抑制剂用于治疗浆细胞疾病的报道。与国外研究不同的是，我们使用维奈克拉的剂量远低于BELLINI研究[2]，且硼替佐米耐药的患者同样取得了较好的疗效。

## 病例与方法

1. 病例：纳入北京大学人民医院血液科自2017年12月至2021年6月收治的17例伴t（11;14）的MM患者进行回顾性分析，患者均接受以维奈克拉为主的方案治疗。该研究是由研究者发起的研究，并已获得北京大学人民医院伦理委员会审批，伦理号为2021PHB211-001。

2. 诊断标准与疗效评估：MM的诊断标准参照2020年MM诊治指南[3]。在新的治疗周期开始前根据IMWG疗效评估标准对患者进行疗效评估，疗效分为：严格意义上的完全缓解（sCR）、完全缓解（CR）、非常好的部分缓解（VGPR）、部分缓解（PR）、疾病稳定（SD）和疾病进展（PD）[4]。ORR为CR率、VGPR率、PR率之和。对完成1个疗程的患者进行疗效评估，治疗不足1个疗程的患者不进行疗效评估。高危细胞遗传学指del（17p）、t（4;14）、t（14;16）。所有应用维奈克拉的患者均进行安全性分析，根据常见不良反应评价标准5.0版（CTCAE 5.0）对入组患者在治疗期间出现的不良事件（AE）进行分级评估。

3. 基线临床特征：收集MM患者的基线临床资料，包括性别、年龄、免疫球蛋白分型、Durie-Salmon（DS）分期[5]、国际分期系统（ISS）分期、修订的国际分期系统（R-ISS）分期[6]、既往MM治疗情况、骨髓细胞形态学检查、流式细胞术检查、细胞遗传学检查（染色体显带分析、荧光原位杂交等）。我院荧光原位杂交技术检测的探针组合为1q21扩增（阈值5％），IgH（14q32）重排（阈值5％），RB1（13q14）缺失（阈值8％），D13S319（13q14.3）缺失（阈值8％），P53（17p13）（阈值8％），具体方法参照文献[7]中的分选方法。所有患者均完成CD138磁珠分选后的荧光原位杂交检查，IgH（14q32）重组阳性患者需要继续检测以下探针：IgH（14q32）/MAF（16q23）、IgH（14q32）/CCND1（11q13）、IgH（14q32）/FGFR3（4p16）。

4. 治疗方案：以维奈克拉为主方案的疗程均为28 d，给药方式为口服，第1个疗程第1天100 mg，第2天200 mg，第3天400 mg，第4～28天为400 mg固定剂量（可根据患者肿瘤负荷及肝功能情况适当调整），从第2个疗程开始予维奈克拉400 mg固定剂量。持续用药直至疾病进展或出现不可耐受的不良反应。出现3～4级血液学或非血液学不良反应时减量至300 mg/d，1例患者因中性粒细胞及血小板减低、肌酐高及高钙血症仅服用100 mg/d×14 d。联合用药方案如下：①VEN+BD方案：维奈克拉剂量及用法同前；硼替佐米1.3 mg·m^−2^·d^−1^，皮下注射，第1、8、15、22天；地塞米松40 mg/d，静脉输注，第1、8、15、22天（75岁以下）或地塞米松20 mg/d，静脉输注，第1、8、15、22天（75岁以上）。②VEN +ITD方案：维奈克拉剂量及用法同前；伊沙佐米4 mg/d 口服，第1、8、15天；沙利度胺100 mg/d，第1～28天，口服；地塞米松20 mg/d，静脉输注，第1、8、15、22天。③维奈克拉+达雷妥尤单抗+硼替佐米+地塞米松：维奈克拉剂量及用法同前；达雷妥尤单抗16 mg·kg^−1^·d^−1^，静脉输注，第1、8、15、22天；硼替佐米1.3 mg·m^−2^·d^−1^，皮下注射，第1、8、15、22天；地塞米松20 mg/d，静脉输注，第1、8、15、22天。其余联合方案均未完整完成一个疗程，包括维奈克拉+硼替佐米+地塞米松+脂质体阿霉素（VEN+PDD方案）及维奈克拉+环磷酰胺+地塞米松+依托泊苷+顺铂（VEN+DECP方案）。

MM患者应用双膦酸盐每月1次治疗骨病，应用阿昔洛韦预防带状疱疹。对于乙型肝炎病毒表面抗原阳性、乙型肝炎病毒DNA阴性患者，治疗过程中不予抗乙型肝炎病毒治疗。对于乙型肝炎病毒表面抗原阳性、乙型肝炎病毒DNA定量≥10^2^患者，治疗的同时予抗乙型肝炎病毒治疗。

5. 统计学处理：运用SPSS 22.0软件进行统计学分析，符合正态分布的计量资料以均数±标准差表示，不符合正态分布的计量资料以*M*（范围）表示。

## 结果

1. 基线数据：本研究共纳入17例伴t（11;14）的MM患者，中位年龄59（46～82）岁，≥65岁患者3例，男9例，女8例。M蛋白分型中轻链型10例，IgG型4例，IgA型1例，IgD型2例。初诊时ISS分期Ⅰ、Ⅱ、Ⅲ期分别为5、7、5例，R-ISS分期Ⅰ、Ⅱ、Ⅲ期分别为5、11、1例。既往治疗一线、二线、三线及以上患者分别为3、6、8例，既往中位治疗线数为2线。5例患者FISH检查结果伴1q21扩增，其余患者除伴t（11;14）外无其他高危细胞遗传学异常。2例患者为外院患者，仅有t（11;14）阳性结果。在有FISH定量数据的15例患者中，t（11；14）中位拷贝数为36％（8％～69％）。2例患者自体造血干细胞移植后复发。16例患者应用蛋白酶体抑制剂（proteasome inhibitor, PI）治疗，15例应用免疫调节剂治疗，14例应用PI+免疫调节剂治疗，14例患者PI耐药，13例患者PI及免疫调节剂耐药。

2. 治疗、疗效及生存情况：17例MM患者中，应用VEN+BD方案者11例，应用维奈克拉单药者2例，余联合方案均为1例。2例患者三系血细胞减低，维奈克拉最大应用剂量分别为100 mg及300 mg，另2例患者分别因经济原因及腹泻应用维奈克拉的最大剂量为300 mg，其余患者均剂量爬坡至400 mg并维持应用。

对应用维奈克拉完成1个疗程的患者进行疗效评估，17例MM患者中共14例可进行疗效评估，3例患者因未完成1个完整疗程而无法进行评估（1例未完成1个疗程即死亡，另2例分别因严重三系血细胞减低及严重腹泻未完成1个疗程）。12例（85.7％）患者获得PR以上疗效，其中血液学CR 1例，VGPR 4例，PR 7例。在应用VEN+BD方案的11例MM患者中，10例可进行疗效评估，获得PR以上患者8例（80.0％），其中血液学CR 1例，VGPR 2例，PR 5例。对PI耐药的14例MM患者中，11例可进行疗效评估，PR以上患者9例（81.8％），其中VGPR 4例，PR 5例。在应用VEN+BD方案且PI耐药的9例MM患者中，有8例可进行疗效评估，PR以上患者6例（75％），其中VGPR 2例，PR 4例。患者详细方案及疗效见表1。

**表1:** 17例应用维奈克拉的伴t（11;14）多发性骨髓瘤患者的临床特征、治疗方案及疗效

例号	年龄（岁）	性别	分型	既往治疗线数	维奈克拉最大剂量（mg）	治疗方案（方案×疗程数）	最佳疗效	t（11;14）比例（％）
1	62	男	λ	二线	300	VEN单药×0.5	无法评估	12
2	67	男	IgG κ	五线	300	VEN+BD方案×8	SD	10
3	56	女	κ	六线	300	VEN×12+ BD×1	VGPR	8
4	59	男	IgG κ	五线	400	VEN+BD×4	PR	91
5	46	男	κ	二线	400	VEN+BD×4+ASCT	PR	68
6	57	女	λ	四线	400	VEN+BD×2；I+VEN+TD×4	PD；SD	31
7	61	女	κ	七线	400	VEN+DECP×5	PR	28
8	55	男	IgA κ	四线	400	VEN+BD×10	VGPR	69
9	61	女	IgG λ	三线	400	VEN+BD×3+ASCT	PR；VGPR	36
10	51	男	κ	二线	400	VEN+Dara×2+allo-HSCT	VGPR；CR	阳性
11	56	男	IgG λ	二线	400	VEN单药×6	VGPR	阳性
12	56	女	IgD λ	二线	400	VEN+BD×4	PR	46
13	71	女	IgD λ	一线	400	VEN+BD×3	PR	58
14	82	女	κ	一线	400	VEN+PDD×0.5	无法评估	21
15	61	男	λ	三线	100	VEN+BD×0.5	无法评估	36
16	59	男	κ	二线	400	VEN+Dara+BD×5	PR	29
17	61	女	λ	一线	400	VEN+BD×3	CR	12

注：VEN：维奈克拉；BD：硼替佐米+地塞米松；I：伊沙佐米；TD：沙利度胺+地塞米松；DECP：环磷酰胺+地塞米松+依托泊苷+顺铂；ASCT：自体造血干细胞移植；Dara：达雷妥尤单抗；PDD：硼替佐米+地塞米松+脂质体阿霉素；allo-HSCT：异基因造血干细胞移植；SD：疾病稳定；VGPR：非常好的部分缓解；PR：部分缓解；PD：疾病进展；CR：完全缓解

截至末次随访时间2021年6月，17例MM患者中位随访9.0（0.5～27.0）个月，失访0例，死亡6例，维奈克拉中位应用周期数为4.0（0.5～12.0）个，维奈克拉中位起效时间为2.0（0.5～4.0）个月。应用VEN+BD方案的11例MM患者中位随访10.0（0.5～27.0）个月，失访0例，死亡3例，其中维奈克拉中位应用周期数为4.0（0.5～12.0）个，维奈克拉中位起效时间为2.0（0.5～3.0）个月。

3. 安全性分析：≥3级AE主要为血小板减少（3例）、感染（2例）、中性粒细胞减少（1例）、腹泻（1例）、周围神经病变（1例），无导致死亡的AE发生，5例患者因PD死亡，1例患者因脑出血死亡（该患者病程中PLT正常）。最常见的血液学AE为血小板减少（7例）、中性粒细胞减少（6例）。常见的非血液学AE为腹泻（5例），恶心、厌食（5例），周围神经病变（3例），感染（2例）及肝功能异常（1例）。随访期内未发生肿瘤溶解综合征及第二肿瘤。

## 讨论

随着MM预后分层的进一步细化及二代测序在MM精细分层中的应用，MM的精准治疗势在必行，BCL-2抑制剂是其中第一个示范性药物。

BCL-2蛋白家族控制固有的凋亡途径，包括促凋亡和抗凋亡蛋白。研究表明骨髓微环境诱导抗凋亡BCL-2家族高表达，从而促进MM细胞的存活。约20％MM患者携带与BCL-2过表达相关的t（11;14）[8]–[10]，且既往研究表明BCL-2抑制剂并非仅对伴t（11;14）的患者有效，对MCL-1/BCL-XL低表达的患者也有显著效果[11]。病例报告显示维奈克拉对伴17p缺失的t（11;14）患者依然有效[12]–[13]。维奈克拉、硼替佐米及地塞米松等药物组合的部分作用机制是后两者通过降低MCL-1的表达促进浆细胞凋亡以增强维奈克拉的抗肿瘤作用。

近年来，以维奈克拉为主的方案治疗RRMM的临床试验取得了令人瞩目的结果。一项Ⅰa期临床试验纳入了66例中位接受5线治疗的RRMM患者，维奈克拉单药的有效率为21％，其中t（11;14）患者的ORR可达40％[14]。在另一项研究VEN+BD方案的Ⅰb期临床试验中，整体ORR达到67％，其中42％疗效≥VGPR。在既往接受1～3线治疗且PI敏感的RRMM患者中，ORR为97％，≥VGPR率为73％[15]。BELLINI研究是一项应用VEN+BD方案的Ⅲ期临床研究，该项研究共纳入291例RRMM患者，均为来那度胺治疗失败且除外PI耐药的患者，该研究的中位随访时间为18.7个月，ORR为78％，中位无进展生存时间为22.4个月，其中t（11;14）阳性患者的ORR为90％。但Ven+BD组的中位OS较VEN+地塞米松组差（*HR*＝2.027）[2]。除MM外，近期法国一项回顾性研究纳入10例复发难治性轻链型淀粉样变性患者，发现单用维奈克拉（400 mg）的ORR可达66.6％[16]。维奈克拉治疗浆细胞白血病的报道基本均为个案报道，但均有良好的结果[17]–[19]。

本研究组自2017年12月开始尝试在伴t（11;14）的浆细胞病患者中应用以维奈克拉为主的治疗方案，本研究共纳入17例伴t（11;14）的RRMM患者，予以维奈克拉为主的化疗方案，ORR为85.7％。应用VEN+BD方案患者的ORR为80％。应用VEN+BD方案且PI耐药患者的ORR为75％。BELLINI试验未纳入对PI耐药的患者，而本研究组应用VEN+BD方案且PI耐药的MM患者ORR可达到75％，证实维奈克拉对PI耐药的MM患者依然有75％的有效率[2]。BELLINI试验中VEN+BD方案组的中位OS较维奈克拉+地塞米松方案组更差，考虑其主要原因为感染事件的发生，有3例患者因感染死亡[2]。本研究患者感染的发生率仅为11.7％，小于BELLIN试验的16％，且没有严重感染导致的死亡事件。本研究的ORR较BELLINI试验低，考虑主要原因为本中心应用维奈克拉的剂量为400 mg，是BELLINI试验剂量的一半；其次，可能与样本量及纳入患者均PI耐药有关。在今后的临床用药中，如患者可表现出较好的耐受性，可以考虑增加维奈克拉的剂量以进一步观察患者疗效是否有进一步提高。

本研究例10为仅血清游离轻链可测型MM，既往应用RVD方案4个疗程，最佳疗效仅为SD，后换用泊马度胺+达雷妥尤单抗+环磷酰胺+脂质体阿霉素+地塞米松方案2个疗程后，受累游离轻链从1 260 mg/L降至777 mg/L，且出现严重肺部感染，来我院后予患者维奈克拉+达雷妥尤单抗，仅半个月疗效就达到VGPR，受累轻链降至59.4 mg/L，并在2个疗程后序贯异基因造血干细胞移植，最佳疗效达到CR。本研究例8既往应用达雷妥尤单抗联合BD方案7个疗程后最佳疗效仅为SD，换用维奈克拉+BD方案后仅2周便达到VGPR并维持至今。

本研究显示以维奈克拉为主的治疗方案整体安全性良好，≥3级AE主要为血细胞减少、感染及腹泻，无治疗相关死亡事件的发生。≥3级血小板减少的发生率与BELLINI试验相当，≥3级中性粒细胞减少、感染及腹泻的发生率较BELLINI试验低，分别为5.9％对18％、11.7％对16％、5.9％对11％。既往维奈克拉在CLL患者中最严重的不良反应为肿瘤溶解综合征，研究发现MM患者对该药的耐受性较好，截至目前仅有2例肿瘤溶解综合征的报道[20]。本研究为回顾性分析，可能会出现不良反应被低估的情况，我们也将在今后的临床工作中进一步观察。

综上所述，本研究是一项在中国进行的单中心回顾性研究，所纳入患者均为伴t（11;14）的以维奈克拉为主治疗方案的MM患者，患者取得了较为理想的疗效和安全性，其长期生存有待更长时间的随访和大样本临床研究证实。同时，本研究也不可避免地存在不足之处，包括样本量小、未测定患者体内BCL-2表达量、前期应用维奈克拉是将其作为晚期患者的姑息治疗、未精确统计全部患者起效时间及起效周期数、回顾性研究本身的局限性等，因此研究结果尚需后续更大样本量及更全面的检测加以完善。
